# Abdominal parietal metastasis on operative scar of gastric adenocarcinoma after R0 resection: An unusual location (about a case)

**DOI:** 10.1016/j.ijscr.2024.110598

**Published:** 2024-11-14

**Authors:** Kouassi Henry Noel AHUE, Moctar KEITA, Kouide Marius GOHO, Israel N'Guessan Saint-Blanc YAPO, Auguste Alexandre ADON, N'Golo Adama COULIBALY

**Affiliations:** a Felix Houphouët-Boigny University, Abidjan, Ivory Coast; b Department of General, Digestive and Endocrine Surgery, Treichville Hospital and University Center, Abidjan, Ivory Coast; c Surgical Emergency Department of the Treichville Hospital and University Center, Abidjan, Ivory Coast; d Abidjan Military Hospital, Ivory Coast; e Cancer Department, Hospital and University Center of Treichville Abidjan, Ivory Coast

**Keywords:** Parietal metastasis, Gastric cancer, Chemotherapy, Abidjan, Ivory Coast

## Abstract

**Introduction:**

Gastric cancer is the fifth most common cancer in Ivory Coast, and is the third cause of cancer death. Parietal metastasis is extremely rare and are distinguished by their relatively poor prognosis with a median survival not exceeding 7 months.

**Observation:**

We report the case of a 73-year-old male patient who presented 5 months after a partial R0 lower polar gastrectomy for gastric adenocarcinoma, a single cutaneous metastasis at the level of the laparotomy incision. The histology of this metastasis was an adenocarcinoma. Multidisciplinary consultation meeting, palliative chemotherapy and metastasis surgery was proposed but with the rapid progression of the tumor disease the patient died 3 months after the diagnosis of the metastasis.

**Discussion:**

Gastric cancer is the fifth most common cancer in Ivory Coast, and is the third cause of cancer death. The most common metastases in patients are the liver, peritoneum and lungs. Parietal metastasis are rare and their frequency is estimated at 4 % in visceral cancers. Anterior abdominal wall metastases have mainly been associated with surgical incision, whether by laparotomy or laparoscopy. Clinical representation is often in the form of dermal or hypodermal nodules of variable size and generally limited number, rapid growth. The diagnosis is made either by carrying out a biopsy or by anatomopathological examination of the surgical specimen. The management is palliative. The prognosis for parietal metastasis is often poor.

**Conclusion:**

Parietal metastasis has a poor prognosis and should always be considered in the face of skin lesions in patients with a history of cancer.

## Highlights

• Parietal metastases from gastric adenocarcinoma are rare.
• The diagnosis is essentially histological.
• The prognosis is very bad.
• The literature concerning its description is sparse and the treatment options are poorly codified.

## 1 Introduction

Gastric cancer is the fifth most common cancer in Ivory Coast, and is the third cause of cancer death [1]. There are multiple pathways of gastric cancer dissemination in metastatic disease. Parietal metastasis are extremely rare and are estimated at 4 % in visceral cancers [2] and are distinguished by their relatively poor prognosis with a median survival not exceeding 7 months [3]. We report the case of a 73-year-old male patient who presented 5 months after an R0 gastrectomy for adenocarcinoma, a single parietal metastasis at the level of the laparotomy incision with rapid progression and the patient died 3 months after the diagnosis of the metastasis. The work has been reported in accordance with the SCARE criteria [4].

## 2 Patient and observation

### 2.1 Patient information

This is a male patient, aged 73, operated on for an adenocarcinoma of the stomach classified pT3N1Mx with lymph node metastases. In a multidisciplinary consultation meeting we decided on peri-operative chemotherapy. The patient returned one month after this decision with gastric stenosis syndrome. Before the gastric stenosis syndrome the surgery was performed without first chemotherapy. It was a 4/5 lower polar gastrectomy with lymph node dissection. Intraoperatively it was a stenosing antropyloric tumor without locoregional invade. There was no secondary location. He benefited from a blood transfusion before and after surgery. The analysis of the operative example shows a well differentiate adenocarcinoma with healthy resection margins and 5 lymph nodes examined. The Operative Follow-Up Were simple. The patient was lost to follow-up for family reasons and consulted 5 months later for a large abdominal mass having started on the upper pole of the laparotomy incision. This mass progressively evolved along the scar. It should be noted that this patient did not benefit from any chemotherapy.

## 3 Clinical results

**Fig. 1:**
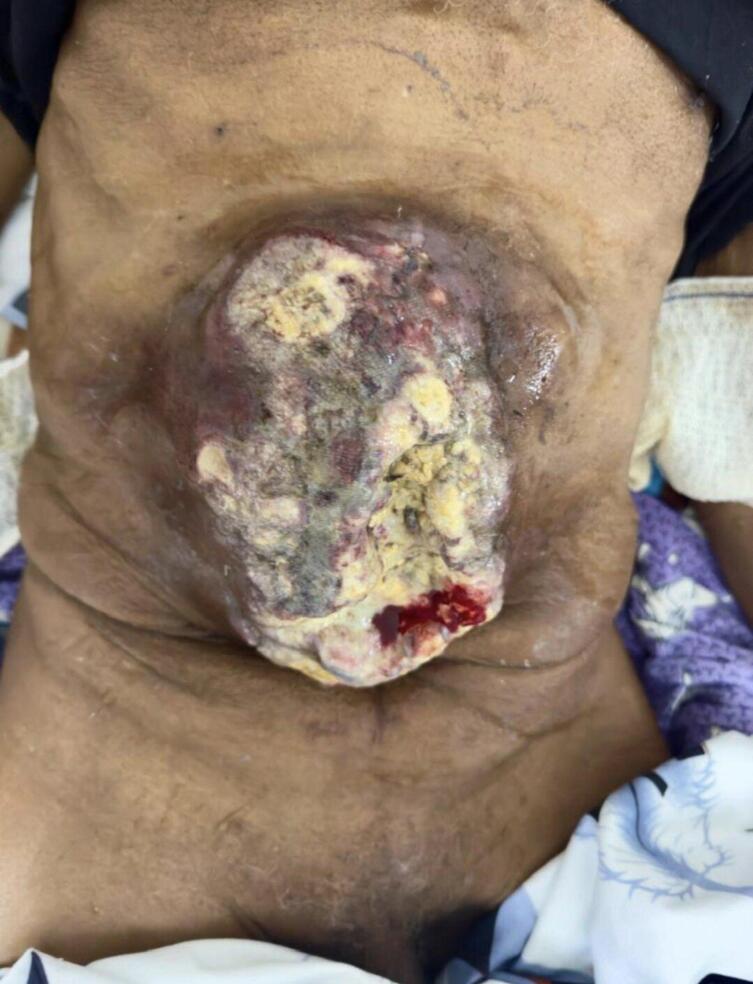
Budding parietal abdominal ulcer tumor.

The clinical examination on admission found a patient with poor general condition WHO 3, emaciated (weight loss of more than 10 %), asthenic, pale conjunctiva, blood pressure at 111/76 mmHg, heart rate at 80 beats per minute, weighing 70 kg and measuring 180 cm, with a temperature of 36.7 degrees Celsius. examination of the abdomen found a budding ulcerative mass measuring 13 cm hypogastrium, oval, with irregular contour and surface, mobile in relation to the deep plane, bleeding easily on contact (Fig. 1). the rest of the abdomen was supple without other anomalies. The patient shows no functional digestive signs.

### 3.1 Diagnostic approach

**Fig. 2:**
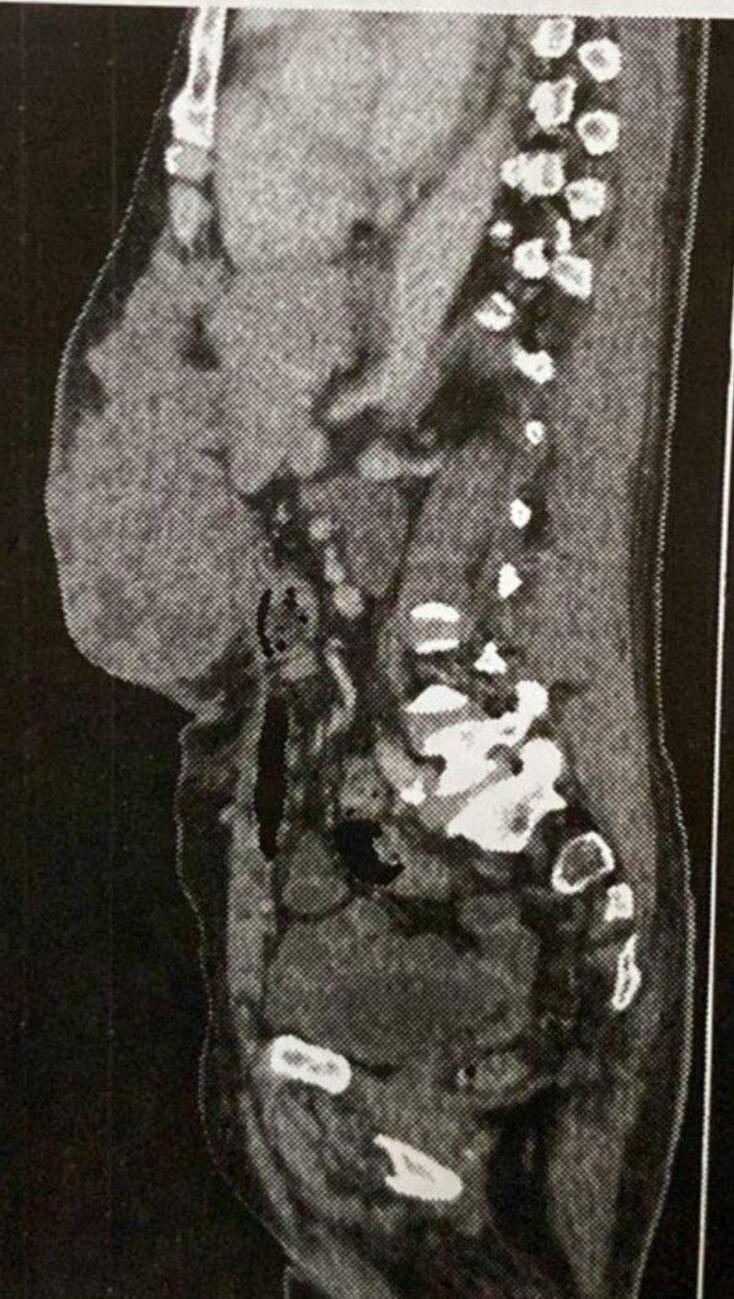
Tumoral parietal process emerging at the level of the white line, it measures 99 mm × 90 mm on an axial section.

An eso-gastro fibroscopy was carried out, showing a normal gastric wall, an abdominal scan describes a tumoral parietal process emerging at the level of the white line, it measures 99 mm × 90 mm on an axial section, is spontaneously hypodense to the muscle increasing after injection of contrast product (Fig. 2). Absence of gastric tumor process. A directed biopsy was carried out, the anatomical pathological examination of which came back in favor of a poorly differentiated tubular adenocarcinoma. As part of the extension assessment, a thoraco abdomino pelvic scan was carried out and did not reveal any other secondary localization. On the biological level, the patient presented anemia at 6 g/dl with hydro-electrolyte disorders, hypoproteinemia, hyperleukocytosis. In a multidisciplinary consultation meeting the decision of palliative chemotherapy associated with possible surgery for excision of the tumor and reconstruction parietal. As the mass progressed, it became more and more hemorrhagic, leading to the death of the patient with severe anemia after 3 months.

## 4 Discussion

The SCARE criteria were used to assess every article [4]. Gastric cancer is the fifth most common cancer in Ivory Coast, and is the third cause of cancer death [1]. There are multiple routes of dissemination of gastric cancer in metastatic disease: lymphatic dissemination (74–88 %), subperitoneal dissemination along the perigastric ligaments, mesentery or omentum, direct invasion into adjacent organs (this is i.e. esophagus 60 %), transperitoneal seeding (53 %), and hematogenous dissemination (i.e. as seen in the rate of liver metastases) [5]. Of all patients with gastric adenocarcinoma, an estimated 26 % have single-site metastases and 13 % have multi-site metastases, the most common of which are the liver, peritoneum, and lungs [6]. Parietal metastasis is rare and their frequency is estimated at 4 % in visceral cancers [2]. Parietal metastasis appears most often during a known neoplasia. In our observation it occurs 5 months after gastrectomy for stomach cancer. In 2021, ELISABETH JACOB found 34 cases of parietal metastases since 1961, testifying to the rarity of this clinical presentation [7]. The mode of propagation is by lymphatic route, by blood route, by contiguity or exceptionally by iatrogenic implantation [8]. Lymphatic metastases are the most common. They are often located near the primary cancer and tend to occur late in the course of the disease. Blood metastases correspond to early dissemination of the cancer. Invasions by contiguity are generally the result of very advanced and neglected cancers. Iatrogenic dissemination can appear after biopsy or evacuation puncture, more exceptionally on laparotopmy scar [8]. Anterior abdominal wall metastases have mainly been associated with surgical incision, whether by laparotomy or laparoscopy [9]. Indeed, the main mechanism suggested would be the direct seeding of neoplastic cells on the anterior abdominal wall during exteriorization of the surgical specimen [10]. The clinical representation is often in the form of dermal or hypodermal nodules of variable size and generally limited number, of rapid growth, eventually stabilizing in their expansion. The surrounding skin is normal or inflammatory; or even ulcerated [11]. Skin metastases are typically painless when they are neither large nor infected. The diagnosis is made either by performing a biopsy or by pathological examination of the surgical specimen [12]. The histological type depends on the type of the primary tumor. In several series adenocarcinoma is the most common hystological type as in our presentation [13]. The therapeutic approach to parietal metastasis is based on good management of the primary tumor if it has been identified. The presence of other metastases added to the skin metastasis, which is the most common case, means that chemotherapy directed against the primary tumor is the only option that allows complete remission to be achieved [14]. Surgery and radiotherapy are often used to treat skin metastases, however no increase in survival has been demonstrated and the aim of these treatments is purely palliative [15]. It has been suggested that surgery could increase the survival of patients with skin metastases from a pulmonary or digestive primary [16]. Adequate palliative treatment of CD includes control of pain, pruritus of bacterial superinfection and in some cases unpleasant odor. If these are numerous and large and cannot be surgically removed [16]. Given the very poor prognosis of this condition and taking into account the metastatic nature of the disease, treatment is generally palliative [3]. The prognosis for skin metastases is often poor with a median survival of 3 to 7 months and an overall survival of 0 % at one year [2]. Survival in our patient was 3 months after diagnosis of metastasis.

## 5 Conclusion

Parietal metastasis is rare and occurs during the evolution of the tumor pathology. The treatment of parietal metastasis remains only palliative, it is based on systemic chemotherapy.

The prognosis is poor. Even though this case has not been successfully treated, it will still be of interest to surgeons, oncologists, general practitioners and family doctors about the possibility of this clinical presentation.

## Author contribution

All authors contributed equally to the article and read and approved the final version of the article.

Kouassi Henry Noel **AHUE**, Moctar **KEITA**, Kouide Marius **GOHO**, Israel N'Guessan Saint Blanc **YAPO**, Auguste Alexandre **ADON**, N'golo Adama COULIBALY, these authors participated in the making and correction of this document. All authors agreed with the publication of the document.

## Consent

Written informed consent was obtained from the patient for publication of this case report and accompanying images. A copy of the written consent is available for review by the Editor-in-Chief of this journal on request.

## Ethical approval

The ethical committee of the Treichville Hospital University center in Ivory Coast gave the agreement to report this case.

## Guarantor

Kouassi Henry Noel AHUE.

## Provenance and peer review

Not commissioned, externally peer-reviewed.

## Registration of research studies

1. Name of the registry: Researchregistry.com – for all human studies.
2. Unique identifying number or registration ID: researchregistry 10762.
3. Hyperlink to your specific registration (must be publicly accessible and will be checked): https://www.researchregistry.com/browse-the-registry#home/.

## Funding

The authors declare they have received no funding for the preparation of this document.

## Conflict of interest statement

The authors report no declarations of interest.
